# An Evaluation of 20 Years of EU Framework Programme-Funded Immune-Mediated Inflammatory Translational Research in Non-Human Primates

**DOI:** 10.3389/fimmu.2016.00462

**Published:** 2016-11-07

**Authors:** Krista G. Haanstra, Margreet Jonker, Bert A. ‘t Hart

**Affiliations:** ^1^Department of Immunobiology, Biomedical Primate Research Centre, Rijswijk, Netherlands; ^2^Department of Immunohematology, Leiden University Medical Center, Leiden, Netherlands; ^3^Department of Neuroscience, University Medical Center, University of Groningen, Groningen, Netherlands

**Keywords:** overview, EU projects, non-human primates, preclinical research, translational research

## Abstract

Aging western societies are facing an increasing prevalence of chronic inflammatory and degenerative diseases for which often no effective treatments exist, resulting in increasing health-care expenditure. Despite high investments in drug development, the number of promising new drug candidates decreases. We propose that preclinical research in non-human primates can help to bridge the gap between drug discovery and drug prescription. Translational research covers various stages of drug development of which preclinical efficacy tests in valid animal models is usually the last stage. Preclinical research in non-human primates may be essential in the evaluation of new drugs or therapies when a relevant rodent model is not available. Non-human primate models for life-threatening or severely debilitating diseases in humans are available at the Biomedical Primate Research Centre (BPRC). These have been instrumental in translational research for several decades. In order to stimulate European health research and innovation from bench to bedside, the European Commission has invested heavily in access to non-human primate research for more than 20 years. BPRC has hosted European users in a series of transnational access programs covering a wide range of research areas with the common theme being immune-mediated inflammatory disorders. We present an overview of the results and give an account of the studies performed as part of European Union Framework Programme (EU FP)-funded translational non-human primate research performed at the BPRC. These data illustrate the value of translational non-human primate research for the development of new therapies and emphasize the importance of EU FP funding in drug development.

## Introduction

A broadly recognized concern that formed the basis of the restructuring of the Life Sciences and Health program funded by the European Commission (EC) is the poor translation of scientific discoveries into effective treatments for patients (1–3). Potential new therapeutics are often target specific and may not react with the homologous target in rodents. Combined with the limited predictive value of rodent disease models for the human disease (4, 5), non-human primates may be the species of choice for preclinical testing. Furthermore, accumulating evidence shows that the aging of the immune system under the influence of chronic latent infections, such as herpesviruses (CMV, EBV), is an important driver of chronic inflammatory disorders *via* mechanisms that are not present in SPF rodents (6). Especially in the field of immune-mediated inflammatory disorders, non-human primates may help to bridge the gap between animal model and patient (5, 7). Non-human primate models’ may also be valuable for reverse translational research into the causes underlying the high attrition of new therapies (8).

Europe houses several primate research centers with the facilities and expertise for preclinical research; the Biomedical Primate Research Centre (BPRC) in Rijswijk, The Netherlands is one of the largest.^1^ Non-human primate research is expensive and requires specialized infrastructure. Academic investigators and small- to medium-sized (SME) biotech companies often have difficulties to cover the high costs for proof-of-concept studies in non-human primates.

The EC has recognized the importance of preclinical non-human primate research and the high costs associated with it. Transnational access (TA) programs to non-human primate research have been installed under various EU Framework Programmes (FP) providing essential funds that helped investigators with the development of new therapeutic entities or with testing new scientific hypotheses.

Research in non-human primates falls under the EU Directive on the protection of animals used for scientific purposes that was entered into force in 2010.^2^ The directive explicitly states that “*to the present state of scientific knowledge, the use of non-human primates in scientific procedures is still necessary in biomedical research*,” but that “*the use of non-human primates should be permitted only in those biomedical areas essential for the benefit of human beings, for which no other alternative replacement methods are yet available*.”

Against these backgrounds, we have analyzed 20 years of EU FP-funded translational research studies in non-human primates performed at the BPRC. In our analyses, we have included 47 studies performed as part of EU FP-funded translational research into the pathogenesis and treatment of disorders caused by the immune system performed between 1996 and 2015.

The results of EU FP-funded studies are often not published within the project period and are thus not visible in the CORDIS database, “the European Commission’s primary public repository and portal to disseminate information on all EU FP-funded research projects and their results in the broadest sense.”^3^ In addition, the results of the preclinical non-human primate research may not be published at all, when study results are inconclusive or negative. By presenting an overview of EU FP-funded TA to non-human primate models for immune-mediated and neurodegenerative preclinical research performed at BPRC, we want to provide insight into these processes. By including unpublished studies in our analysis, we provide a different view on the contribution of non-human primate research to the development of new therapies as compared to an analysis of the drug registration files (9, 10). Such analyses underestimate the value of non-human primate preclinical research, as drugs that are not registered because of failure in non-human primate studies are usually not included.

## Overview of 20 Years Non-Human Primate Transnational Access Programs

The BPRC has been involved in EU FP-funded preclinical research in non-human primate models for chronic and infectious diseases for more than 20 years. The first TA program started under the third FP: Biomedical Primate Research Centre: non-human primates as models for human biology and disease (BPRC), CORDIS database reference CHGE940071. Together with two EU Concerted Actions (references MR4*0276 and BMH11531), this multiannual program has resulted in a number of publications (11–23). However, information on studies that were performed as part of these programs, but were not published, is lacking. We therefore focus our review on the period between 1996 and 2015. In this period, BPRC has hosted five EU FP-funded TA programs and has participated in four EU FP-funded consortia dedicated to preclinical non-human primate research on chronic, degenerative, and infectious diseases (Table 1).

**Table 1:** **Overview of transnational access programs and dedicated consortia**.

Acronym	Reference CORDIS	TA/consortium	Framework programme	Period	Title
CFIT	BMH4960127	TA	4	1996–1998	EU Centralized Facility for ImmunoTherapy Evaluation (24)
BPRC-LSF	FMGE950024	TA	4	1996–1999	BPRC large scale facility: non-human primates as models for human biology and disease (24)
PCDD	HPRI-CT-2001-00150	TA	5	2001–2004	Non-human primates as Preclinical Models Facility of Chronic and Degenerative Diseases in humans (25)
PRIMOCID	26155^a^	TA	6	2006–2010	Primate models of chronic and immune-based diseases
PRIMOCID-II	262443^a^	TA	7	2011–2015	Primate models of chronic and infectious diseases-II^b^
–	BMH4972131	Consortium	4	1997–2000	Targeted anti-CD40 monoclonal antibodies for treatment of multiple sclerosis
EUPEAH	QLRI-CT-2002-02758	Consortium	5	2003–2008	Glucocorticoid hormone programing in early life and its impact on adult health
TRIAD	281493	Consortium	7	2012–2014	Tolerance restoration in autoimmune diseases by selective manipulation of the CD28 costimulatory pathway^c^
PRIMOMED	606084	Consortium	7	2014–2015	Use of PRIMate MOdels to support translational MEDicine and advance disease modifying therapies for unmet medical needs^d^

*^a^PRIMOCID programs were part of the Research Infrastructures of EUPRIM-Net (http://www.euprim-net.eu/home.htm) under FP 5 and FP6*.

*^b^http://www.bprc.nl/en/eu-sponsored-transnational-access*.

*^c^https://www.triad-cd28.eu/*.

*^d^http://www.primomed.fp7sme.eu/*.

In the 1980s and 1990s, treatment for prevention of organ transplant rejection was a major driver of immunosuppressive drugs development. For decades, (non-human primate) organ transplantation has been the favored model for immunosuppressive drug evaluation, as the target of the immune response is known, namely, the grafted organ (kidney) or tissue (skin). Development of models for autoimmune-mediated inflammatory disease (AIMID) has gradually replaced transplantation as the model of choice for immunosuppressive drug discovery. Often, the efficacy of a new drug needs to be established first in a relevant model, and new treatments do not need to be developed for each specific disease. The available non-human primate models of AIMID, collagen-induced arthritis (CIA), and experimental autoimmune encephalomyelitis (EAE) are now used as prototype models for a broader spectrum of (auto)immune-mediated diseases in the human population. This seems justified, as recent genome-wide association studies show that pathways of immune activation are common to a wide range of diseases (26). Many treatments are at first instance indicated for diseases affecting a large number of patients, such as rheumatoid arthritis (RA). Drugs successfully used for high prevalence indications are subsequently tested for diseases affecting smaller numbers of patients or for indications where the relevance of the target in the disease is less well established.

Projects submitted for execution under the TA were always reviewed by a User Selection Panel, which consisted of international experts in the fields of research covered by the TA and principal investigators from the BPRC. Criteria for selection were scientific relevance and quality, the necessity for testing in non-human primates and the likelihood that research objectives could be reached. Selected projects were subsequently submitted to the institute’s ethics committee for review of the experimental design and procedures. The complete costs for purchase of genetically typed monkeys, housing and care, and all experimental procedures of elected projects were covered by the TA or the consortia.

Research topics for TA programs were obviously a reflection of the societal challenges and public interest of their times. For the “EU Centralized Facility for ImmunoTherapy Evaluation” (acronym: CFIT) program, which ran from 1996 to 1998 [see Table 1 (24)], two focuses were identified by the User Selection Panel, namely (1) interference with immune activation, with a strong focus on the induction of immune tolerance, and (2) application of somatic gene therapy and/or stem cells in chronic inflammatory diseases. In addition, part of the activities was aimed at the development and improvement of non-invasive methods to assess disease severity and the development of *in vitro* parameters for prediction of the *in vivo* outcome of immunotherapy.

The follow-up TA program, “Non-human primates as Preclinical Models Facility of Chronic and Degenerative Diseases in humans” (PCDD), was advertised as a program to initiate a European Immune Tolerance Network (25). Submitted projects were in line with this ambition, indicating that within the EU research community indeed the consensus was that immune tolerance was the ultimate aim for the prevention of organ graft rejection or possibly even for (auto)immune diseases. Intervention in immune activation has remained an important topic over the years. This was also the aim of three of the four dedicated consortia (Table 1) in which BPRC has participated. Immunosuppression is the next best option after disease prevention and/or cure.

In the two subsequent PRIMOCID programs, non-human primate somatic gene therapy research was no longer in fashion and stem cell therapy became a hot topic. The focus of the two PRIMOCID programs shifted further toward understanding disease mechanisms and to apply more refined means of immunosuppression, by interfering with single pathways. The aim of establishing immune tolerance has been gradually left, among others due to observations in monkeys and humans that the pathogen-educated primate immune system may be relatively resistant to tolerance induction strategies (5, 27).

The rationale to include the four EU FP-funded consortia in which BPRC participated, stems from the fact that these consortia had similar aims as the TA programs, the only difference being that BPRC was not the coordinator. The consortia “anti-CD40 in multiple sclerosis,” TRIAD, and PRIMOMED were all aimed at the development of immunosuppressive therapies in non-human primate EAE and CIA models. Within PRIMOMED, there was also a project testing a new therapy for neurodegenerative diseases, which used the MPTP-induced marmoset model of Parkinson’s disease. The aim of EUPEAH was to establish the impact of pre-natal dexamethasone exposure on health parameters in adulthood.

## Achievements

In the period between 1996 and 2015, a total of 38 *in vivo* EU FP-funded studies were executed in TA programs (see Table 2). From 35 of the projects, the original applicant data could be analyzed. One user obtained access twice (in two different programs, on two different models, 7 years apart), resulting in 34 different users. Of those 34 users, 25 were male, 9 were female, and they originated from 12 different EU Member States or associated countries (associated countries are eligible for EU FP funding).

**Table 2:** **Overview of all fully executed *in vivo* studies under the indicated EU FP per disease model and publications**.

	Total # studies		EAE	CIA	Kidney Tx	PD	Infectious diseases	Other
	Executed	Accepted/published						
CFIT	12	8	2 (28)	3 (29–31)	5 (32–35)			2 (36)
BPRC-LSF	3	3	3 (37–39)					
PCDD	7	3	2 (40, 41)	2	1			2 (42)
PRIMOCID	9	5	2 (43, 44)			2^a^ (45)		5 (46, 47)
PRIMOCID-II	7	3	2 (48, 49)	1			3^a^	1 (50)
Dedicated consortia	9	6	7 (28, 51–54)	1 (55)		1^a^		
Total	47	28	18	7	6	3	3	10

*^a^Manuscript(s) in preparation*.

Nine *in vivo* EU FP-funded studies were executed at BPRC as part of consortia coordinated outside the BPRC. A total of 47 EU FP-funded studies included in this analysis have resulted in 28 peer-reviewed papers reporting primary data. One paper contains data obtained from two studies (28), and two papers present primary data from one study (29, 30). The substantial delay between publication of papers and the closure of the projects is exemplified by the fact that four publications are still in preparation, with one paper reporting data collected under the PRIMOCID-I program, which was closed in 2010 (Table 2).

The effects of the indicated foci of each of the programs are reflected in the list of publications. The focus in CFIT on interference with immune activation/tolerance is evidenced by references (28, 32–36) and the focus on somatic gene therapy/stem cells by (29–31). The development and improvement of non-invasive methods has resulted in three additional publications (20, 56, 57). The focus of the PCDD program on tolerance induction is unfortunately not reflected in publications, illustrating the already mentioned difficulty in tolerance induction in the pathogen-educated primate immune system. Four of the seven studies have remained unpublished, of which two were aimed at tolerance induction. The two subsequent PRIMOCID programs did not have predefined foci and were therefore a reflection of the general interest of the research community. In one study, the capacity of human induced pluripotent stem cells (iPS) to repair MS-like brain lesions in marmosets was tested, resulting in a publication (48). The second study describes a pilot study to set up an Alzheimer’s disease model in marmosets (50). Other research themes were understanding disease mechanisms and refinement of immunosuppression, yielding four publications (43–46, 49).

**Figure 1:**
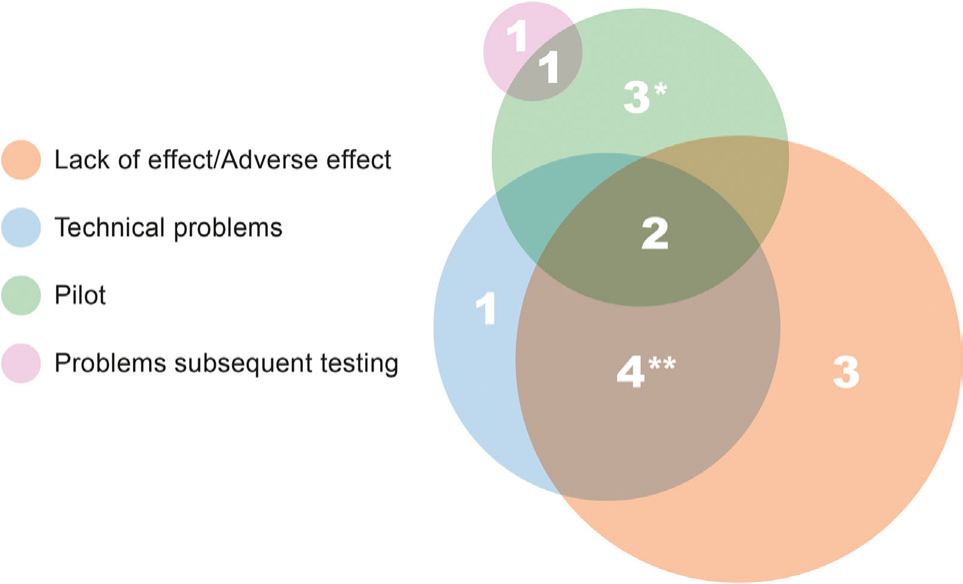
**Causes and/or reasons underlying unpublished studies**. The size of the circle reflects the total number of studies in that category. *Results of one pilot study referred to in review paper (58), and results from the second pilot study are mentioned on SME website (http://www.s-target.com/). **Results of one study published only in a non-peer-reviewed paper (59).

The results of 15 studies were not published as peer-reviewed papers. Figure 1 illustrates the complex interplay of causes for studies not resulting in the publication. The most common reason is absence of a significant beneficial effect, or sometimes even a detrimental effect of the treatment/therapy under study (nine studies). Non-human primate studies are usually small sized and insufficiently powered for detecting small effects or significant effects only in a part of the animals. When a study is then also hampered by technical problems (Figure 1), firm conclusions can often not be drawn, which precludes publication in the peer-reviewed literature. Two reasons underlying five additional unpublished studies are that four studies were designed as pilot studies, and one of them, together with the second study failed in follow-up *in vivo* testing. The unpublished pilot studies were all performed during the first four TA programs. It should be noted that unpublished studies are always accounted for in project reports to the EC.

Publication of negative studies is a hotly debated issue. We strongly believe that important lessons can be learned from negative results, providing that the study was executed without technical problems. It is well possible that the targeted process may be less relevant in a primate model than in lower species. We believe that this is the essence of translational research (7). Studies involving non-human primates are often the end-stage of a long development process, where the candidate therapy has passed several selections. It is important that the reason why a treatment fails at a late development stage is examined and that results are published. The lack of publishable results does not necessarily mean that the study was not valuable. Often the absence of adverse effects, even when positive effects of the therapy/treatment were not obtained, will motivate researchers to further develop the drug or choose another disease where the targeted process may be more relevant.

One of the recommendations of the EUPRIM-Net II funded workshop on Alternative methods for the use of non-human primates in biomedical research was that negative results should be published (60). The EC also strongly advocates public dissemination of the results of funded projects in grant agreements. To cover the costs of open access publication for studies performed under FP7, the EC has installed a support program.^4^ BPRC always requires that researchers of submitted projects are willing to publish the results. The reality is that mandatory publication of negative or inconclusive results would pose a barrier to participate in TA programs.

## Spin-Off

Although most studies were aimed at the efficacy evaluation of new therapies, the spin-off of these studies has reached further than the immediate study aims. Body fluids, tissues, organs, and/or cells collected from *in vivo* studies, EU FP-funded and privately funded, are frequently used at a later stage for *ex vivo* analyses. These analyses drive further investigations into pathogenic pathways. Examples of such analyses are the description of anti-vimentin antibodies in kidney transplantation (61), a still unresolved problem in transplant patients; description of the lack of association of FOXP3 as a regulatory marker in non-human primate transplantation (62); evaluation of IL-1β expression in brain tissue of rhesus macaques with EAE, compared to in MS patients (63), and other descriptive analysis of larger sets of tissues obtained during various studies (64, 65).

Furthermore, results from *in vivo* studies may be used to develop or optimize biomarkers for disease (18, 19), pathological hallmarks, and/or mechanisms (66–68). During the CFIT program, one of the aims was to develop non-invasive diagnostic parameters, which has resulted in a number of important developments for *in vitro* and *ex vivo* analysis (20, 56, 57). These refinements are implemented in subsequent studies.

Starting under the first TA program under FP3, a new model for multiple sclerosis has been developed, EAE in common marmosets (18, 23). The model has been extensively refined throughout subsequent TA programs and used for preclinical efficacy testing (28, 37, 40, 43, 44, 49, 51–53). The close clinical and pathological approximation of the model to the human neurological disease on which it had been projected (multiple sclerosis) has been recognized. This is evidenced by the fact that the model was also the model of choice for preclinical efficacy testing outside EC-funded research (69–73).

## Translational Value of Executed Projects

To answer the question if EC funding of preclinical non-human primate research helps to bridge the gap between drug discovery and drug prescription, a number of issues need to be considered. Some of the projects were submitted by academic researchers, merely to investigate pathogenic mechanisms or the clinical potential of therapies that are not (yet) applicable for human use (29–31, 36–41, 43, 49, 51), and other projects were submitted by medium-sized Pharma companies. Whether development of such drugs would have continued also without access to the TA, cannot be established.

The translational value of the 15 unpublished studies is variable. As discussed, two studies failed in follow-up testing, and the studies were not able to signal the subsequent problems. From three other studies, the drug under investigation entered the TA programs *via* other routes as well, and that drug is still a candidate for clinical testing. One reason as a cause for a study not being published in a peer-reviewed paper is its success for the SME. In the case of one unpublished pilot study (Figure 1), the SME continued with the research and provided proof of therapeutic principle in an efficacy study. The technology was licensed to a pharmaceutical company.^5^ This leaves another nine studies, all for unique drugs or cell therapies, which have not resulted in any economic spin-off.

However, most certainly, EU FP funding has helped to speed up the development of a number of drugs or therapies, although it is still too early for most of them to have reached the clinic. Below illustrative examples are listed.

The clinical development of anti-CD40 monoclonal antibody as immunosuppressive treatment has benefited from several EU FP-funded programs. In its various forms, and under its various owners, several papers were published, with (23, 52, 53, 59) and without (74–77) EU funding. Currently, a clinical grade deimmunized version of the tested antibody is under development by FF Pharma, and phase I clinical trials have been successfully completed.^6^

The proprietary anti-CD28 monoclonal antibody (54, 55) is currently in phase I clinical trials.^7^ An international EU FP-funded consortium was formed, to advance this therapy toward the clinic, and this has been very successful.

Anti-CD20 monoclonal antibodies have been used in patients already, but its mechanism of action had not yet been fully resolved. The mechanism has been studied in one EC-funded study (44), as well as privately funded studies (73, 78, 79).

Amgen has bought Micromet, the company that developed the anti-IL2 receptor monoclonal antibody developed (46). Whether this antibody is still under development by Amgen has not been disclosed on the AMGEN website.

The impact of a posttranslational modification of IgG4 molecules exchanging half-molecules (one heavy chain with its attached light chain), so-called Fab arm exchange, was first described in an EU FP-funded study in the rhesus monkey model for myasthenia gravis (42). This discovery has impacted research with therapeutic monoclonal antibodies in many ways, and nowadays, IgG4 antibodies are often stabilized to prevent unwanted Fab arm exchange (80). Genmab exploits this principle to generate bispecific antibodies.^8^

## Conclusion

In the period of 20 years spanning this review, 47 studies were performed with EC funding. These studies are often a reflection of the interest of the research community of their times. Important for determining the value of non-human primate research in drug development is both the number of studies with positive findings and negative findings. The 47 studies have resulted in 28 peer-reviewed papers, with 4 more papers in preparation, mostly describing important new findings, but in 1 case, negative results were also published (32). Also, more then 10 papers describing *ex vivo* analyses of materials obtained during these studies have been published. Fifteen studies did not result in a publication, primarily because of absence of a beneficial effect. Although we are contractually bound not to disclose details of these studies, this review enables us to picture the complex interplay of causes that prohibit the publication of studies. The most obvious one is the lack of positive results, but additional risk factors are technical problems and the pilot type of studies. Technical problems cannot always be avoided, but the inherent responsibilities resulting from EC funding for TA, dictate that funding should be directed toward established models, with a clear testable hypothesis.

Another possible cause for the absence of an effect may of course also indicate that the drug is just not effective and the development for human use may be reconsidered. For these cases, a database of negative results would certainly benefit the value of the money spent.

The importance of EU FP funding is undisputed, as 95% of these studies could not have been performed without it. It has lead to many important findings, but within this period of 20 years, it has not (yet) resulted in drugs being available for patients.

## Author Contributions

KH collected and analyzed the data and wrote the article. MJ and BH contributed to the collection of the data and were critically involved in the writing of the article.

## Conflict of Interest Statement

The authors declare that the research was conducted in the absence of any commercial or financial relationships that could be construed as a potential conflict of interest.

## Footnotes

^1^www.bprc.nl.

^2^http://eur-lex.europa.eu/eli/dir/2010/63/oj.

^3^http://cordis.europa.eu/projects/home_en.html.

^4^https://postgrantoapilot.openaire.eu/#home.

^5^http://www.s-target.com/.

^6^http://www.ffpharma.com/2014/index.php/clinical-trials/pg102-03.

^7^http://ose-immuno.com/en/portefeuille-de-produits/fr104/.

^8^http://www.genmab.com/duobody/technology.
